# The zinc finger of DNA Ligase 3α binds to nucleosomes via an arginine anchor

**DOI:** 10.21203/rs.3.rs-6033068/v1

**Published:** 2025-03-10

**Authors:** Bennett Van Houten, Ashna Nagpal, Matthew Schaich, Tyler Weaver, Vera Roginskaya, Annahita Sallmyr Sallmyr, Liam Leary, Bret Freudenthal, Alan Tomkinson

**Affiliations:** University of Pittsburgh; University of Pittsburgh; University of Pittsburgh School of Medicine; University of Kansas Medical Center; University of Pittsburgh; University of New Mexico Health Sciences Center; University of Pittsburgh; University of Kansas Medical Center; University of New Mexico Comprehensive Cancer Center

**Keywords:** Single-molecule fluorescence microscopy, nuclear extract, base excision repair, LUMICKS C-trap, nucleosome core particle

## Abstract

Ligation of DNA single strand breaks is critical for maintaining genome integrity during DNA replication and repair. DNA Ligase III (LIG3α) forms an important complex with X-ray cross complementing protein 1 (XRCC1) during single strand break and base excision repair. We utilized a real time single molecule approach to quantify DNA binding kinetics of Halo-tagged LIG3α and XRCC1-YFP from nuclear extracts on long DNA substrates containing nicks, nucleosomes or nicks embedded in nucleosomes. LIG3α displayed higher affinity for nicks than XRCC1 with the LIG3α catalytic core and N-terminal zinc finger (ZnF) competing for nick engagement. Surprisingly, compared to single strand breaks in naked DNA, LIG3α bound even more avidly to an undamaged nucleosome reconstituted on the 601-sequence, with binding dependent on two arginine residues in the N-terminal ZnF. These studies reveal insights into nick detection and identify the role of a novel arginine anchor in LIG3α for engaging nucleosomes.

## Introduction

DNA ligases restore breaks in DNA phosphodiester bonds to maintain genome integrity. These breaks can be generated during DNA replication and repair as well as breaks resulting directly by DNA damaging agents. Human cells contain three forms of mammalian DNA ligases, I, III and IV. Each of these enzymes have similar catalytic regions, including a DNA binding domain (DBD) and a catalytic core (CC) composed of nucleotidyl transferase (NTase) and oligonucleotide/ oligosaccharide-fold binding domains (OBD) ^1–8^. While deletion of each of the *LIG* genes results in embryonic lethality in mice, it was not possible to establish cultures of embryonic fibroblasts from the *LIG3* null embryos in contrast to *LIG1* and *LIG4* null embryos (Bentley et al., 1996; Bentley et al., 2002; Frank et al., 1998; Puebla-Osorio et al., 2006). Subsequently it has been shown that the *LIG3* gene is essential for cell viability under normal growh conditions because it encodes the only mitochondrial DNA ligase in addition to a nuclear version by alternative translation initiation ^9–11^.

The nuclear and mitochondrial versions of LIG3α both lack a nuclear localization signal (NLS). Nuclear LIG3α gains entry into the nucleus by forming a complex with the DNA repair scaffold protein, XRCC1 which has an NLS ^5,12–16^ whereas a mitochondrial targeting sequence within the extra N-terminal region of mitochondrial LIG3α directs this protein to mitochondria where it functions to maintain the mitochondrial genome ^10,17,18^. Notably, disruption of the interaction between the C-terminal BRCT domains of LIG3α and XRCC1 markedly reduces the cellular LIG3α levels. This presumably reflects the role XRCC1 in stabilizing nuclear LIG3α, whereas LIG3α function in mitochondria is independent of XRCC1 ^10,17–20^. Within the nucleus, the best characterized functions of LIG3α and XRCC1 are in the base excision repair (BER) and DNA single-stranded break (SSB) repair pathways ^5,21^. It is likely that these pathways play a critical role in ensuring the viability of differentiated cells since abortive ligation, defects in an interaction between LIG3α and Fused in Sarcoma (FUS), as well as inherited mutations in *XRCC1* have been linked with neurodegeneration ^22–24^. LIG3α and XRCC1 also act in a backup repair mechanism when either of the major DNA double-strand break repair pathways are defective ^5,25–28^, and during the semi-conservative DNA replication via an alternative Okazaki-fragment ligation pathway ^29–32^.

In single strand break repair, the broken sugar-phosphate backbone is initially recognized by PARP1, which is activated to synthesize chains of poly(ADP-ribose) (PAR) that can be covalently linked to proteins, including PARP1 itself ^33,34^. PAR recruits XRCC1 and associated repair proteins, including LIGα to the damage site ^35–37^. Interestingly, the central BRCT of XRCC1 has separate binding sites for PAR and DNA, both of which are involved in the recruitment and retention of XRCC1 at break sites ^38^. In BER, the combined action of DNA glycosylases and AP endonuclease generate a single strand break that is converted into a ligatable nick by Polb. It has been suggested that repair intermediates are sequentially passed to BER enzymes with the XRCC1 scaffolding protein facilitating these hand-offs through interactions with the enzymes including LIG3α ^39^. While PARP1 has been implicated in BER, it’s precise role in processing intermediates is not entirely clear. However, PARP1 clearly contributes to BER when the flux through the initial stages of the pathway exceeds the DNA synthesis and ligation steps ^20,40^.

LIG3α differs from the other ligases as it contains an N-terminal Zinc finger (ZnF) motif that is highly related to the first two zinc finger motifs at the N-terminus of PARP1^41^. The LIG3α ZnF appears to function as a single strand break sensor both in the presence and absence of activated PARP1 ^42^. Biochemical and biophysical studies revealed that the LIG3α ZnF together with the DBD from a single strand break binding module that is insensitive to the break structure whereas the Ntase and OBD form a second single strand break binding module that preferentially binds to ligatable nicks ^40^. This led to the proposal of a “jackknife” mechanism ^6,43^, in which the ZnF-DBD first senses the nick but is then displaced at a ligatable nick by the catalytic core (CC).

In contrast to biochemical and biophysical studies characterizing the interaction of LIG3 with naked DNA, there have been relatively few studies examining how LIG3α engages with intact and nicked DNA packaged into nucleosomes ^44–46^. Interestingly, it has been shown that LIG3α binds to condensed chromatin during metaphase ^47^, and that both LIG3α and XRCC1 associate with undamaged nucleosome core particle (NCPs) ^4^. While it has been suggested that LIG3α/XRCC1 disrupts the nucleosome in order to ligate DNA nicks ^45^, more recent studies indicate that transient unwrapping of DNA, particularly in the regions where DNA enters and exits the nucleosome unwrapping, enables LIG3α/XRCC1 to join DNA nicks ^44,48^. Here we have used single molecule kinetics to examine the binding of tagged versions of XRCC1 and LIG3α in nuclear extracts with intact and nicked DNA in the absence or presence of NCP. These experiments characterized the on and off rates of LIG3α and XRCC1, their order of assembly and disassembly, and how these proteins identify DNA nicks in dsDNA and chromatin. Importantly, using this single-molecule approach, we discovered that the LIG3α ZnF domain utilizes two arginine anchor residues (R62/R64) for binding to undamaged NCP’s.

## Results

### Binding kinetics of LIG3α-XRCC1 on a λ DNA substrate containing eight observable 3’-OH- 5’-phosphate nicks using SMADNE.

**Figure 1:**
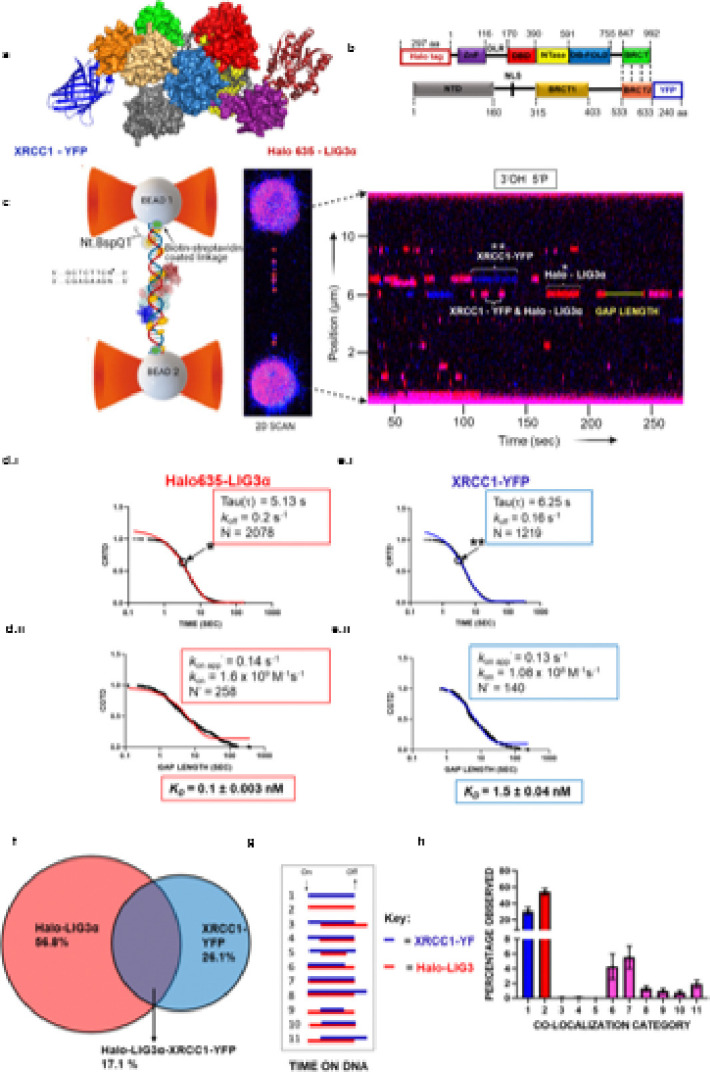
SMADNE analysis of Halo 635-LIG3α-XRCC1-YFP on eight observable nicks in λ DNA. **(a)** A representative structural model of heterodimeric complex model of N-terminal tagged Halo635 LIG3 α and C-terminal YFP tagged XRCC1 bound to nicked DNA (PDB codes 3L2P and 3K77) using Alphafold3, for clarity the disordered regions were removed. **(b)** Domain structures of LIG3 α (nuclear) bound to C-terminal BRCT2 domain of XRCC1 via BRCT domain, forming a heterodimeric complex. **(c)** A schematic 3D model of workflow showing DNA-binding interactions of heterodimeric complex model of N-terminal tagged Halo635 LIG3 αand C-terminal YFP tagged XRCC1 bound to nicked λ DNA digested with nicking enzyme Nt.BspQI generating eight observable ligatable nicks. The substrate (48.5 kb nicked DNA) then gets attached between two polystyrene beads, trapped in the optical tweezers shown as a 2D scan image captured with the binding events for Halo 635-LIG3α-XRCC1-YFP followed by real time kymograph mode at 10 pN tension, where X-axis represents time(sec), and the Y-axis shows the position(μm). **(d)** Cumulative residence time distribution (CRTD) and cumulative gap time distribution (CGTD) analysis for Halo 635-LIG3αbinding nicked DNA, with a single exponential fit shown in red. Asterisks indicate a single binding event occurring in real time. **(e)** Cumulative residence time distribution (CRTD) and cumulative gap time distribution (CGTD) analysis for XRCC1-YFP binding nicked DNA, with a single exponential fit shown in blue. Asterisks indicate a single binding event occurring in real time. **(f)**The percentage of events that were Halo 635-LIG3α alone in red and XRCC1-YFP alone in blue, or colocalized together in the middle as pink. **(g)** 11 possible interactions for Halo 635-LIG3α as red bars and XRCC1-YFP as blue bars binding on DNA respectively. **(h)** The distribution of the 11 categories for Halo 635-LIG3αand XRCC1-YFP binding nicked λDNA. Error bars represent the SEM of fourteen experiments.

We recently developed and validated the SMADNE approach (Single-Molecule Analysis of DNA binding Proteins from Nuclear Extracts) ^49^, which utilizes fluorescently tagged DNA-binding proteins expressed in nuclear extracts to study real time DNA binding dynamics at the single molecule level using a LUMICKS C-trap. In this experimental setup, we designed a structural model of heterodimeric complex of N-terminal tagged Halo635 LIG3 α and C-terminal YFP tagged XRCC1 bound to nicked DNA (PDB codes 3L2P and 3K77) using Alphafold3 (Fig. 1a) and a domain structure of LIG3 α (nuclear) bound to C-terminal BRCT2 domain of XRCC1 via BRCT domain, forming a heterodimeric complex (Fig. 1b). The optical tweezers in the LUMICKS C-trap were used to trap streptavidin coated polystyrene beads. By flowing in biotinylated λ DNA substrate containing eight observable ligatable nicks, one strand of DNA is then strung up between the beads (Fig. 1c, left panel, see Material and Methods). Nuclear extracts expressing fluorescently tagged LIG3α with HaloTag conjugated to Janelia-Fluor 635 dye on the N-terminus and XRCC1 fused to YFP at its C-terminus (Fig. 1a & b, **Fig. S1 & S2**. see Material and Methods) were diluted 1:10 in binding buffer (20mM HEPES pH 7.5, 150mM NaCl, 1mM DTT, 5% glycerol, 0.5mg/ml BSA,1mM trolox) and then flowed into the LUMICKS C-trap flow cell containing the tethered λ DNA. Flow was stopped, and a 2D confocal image was generated to monitor binding of these proteins to the DNA (Fig. 1c, middle panel). We then collected kymographs, where the Y-axis represents the position on the DNA and the X-axis represents time, in 1-dimensional scanning (kymograph) mode at 10 frames per second using the nicked λ DNA (Fig. 1c, right panel). These kymographs revealed HaloTag635-LIG3α binding events as red fluorescent time streaks, XRCC1-YFP binding events as blue fluorescent time streaks, and the simultaneous binding of LIG3α and XRCC1 as pink fluorescent time streaks. Using the SMADNE approach, we calculated the binding lifetimes (Tau) and *k*_off_ using CRTD (cumulative residence time distribution) analysis (Fig. 1d.i and e.i) of all the binding events. Binding event durations were best fit to a one phase exponential decay, yielding lifetimes for LIG3α, Tau = 5.13 sec (± 0.02), and XRCC1, Tau = 6.25 sec (± 0.03) (Fig. 1d.i for LIG3α & **e.i** for XRCC1). By measuring the gap times (the spaces between events at the same DNA position) and fitting to a CGTD (cumulative gap time distribution) curve, a *k*_on app_ for LIG3α and XRCC1 could be determined (Fig. 1d. ii for LIG3α & Fig. 1e. ii for XRCC1). Using the *k*_off_, *k*_on app_, and the protein concentration in the flow-cell (see Materials and Methods and S1C), we also determined equilibrium dissociation constant (*K*_*D*_) of LIG3α and XRCC1 for the nicked DNA substrate. This analysis revealed that LIG3α binds the nicked DNA substrate with a *K*_*D*_ = 0.1 ± 0.003 nM, whereas XRCC1 binds to the nicked DNA substrate with a *K*_*D*_ = 1.5 ± 0.04 nM (see Table 1). Together, our data indicates that both LIG3α and XRCC1 bind the nicked DNA substrate with high affinity, although XRCC1 binds with a ~ 15-fold weaker affinity than LIG3α (see Table 1).

Surprisingly, while LIG3α and XRCC1 sometimes colocalize (17.1%), most of the events were single binding events of LIG3α (56.8%) or XRCC1 (26.1%) as shown in Fig. 1f. Thus, although it has been assumed that nuclear LIG3α functions as an obligate heterodimer with XRCC1, our studies indicate that, at the concentrations used in these experiments (0.1–2 nM) (see calibration curve in **Fig. S1c**), a significant fraction of these proteins are acting independently. In addition to the two single binding events, there are nine possible overlapping binding events with unique orders of assembly and disassembly (Fig. 1g). Using a co-localization script (see Material & Methods) to classify these 11 different types of binding events, we found that the most common colocalization type was category 7, in which both LIG3α and XRCC1 arrive and dissociate together. In addition, binding events in which LIG3α and XRCC1 arrive together (categories 6–8) predominate following by binding events in which LIG3α arrives first (categories 8–11). Colocalization categories 3–5, in which XRCC1 arrives first were very rare. Thus, in accord with their binding affinities, it is evident that LIG3α is driving the overlapping binding events, binding first or simultaneously with XRCC1 at the nick (Fig. 1h, see video supplemental Movie 1 for a working model). However, when the two proteins co-localize, their average lifetime of both proteins together on the DNA was only 0.7s, suggesting the BRCT interaction between the two proteins is weaker than the affinity of a nick by LIG3α (**Fig. S2**).

### Visualizing nick sealing over time

**Figure 2:**
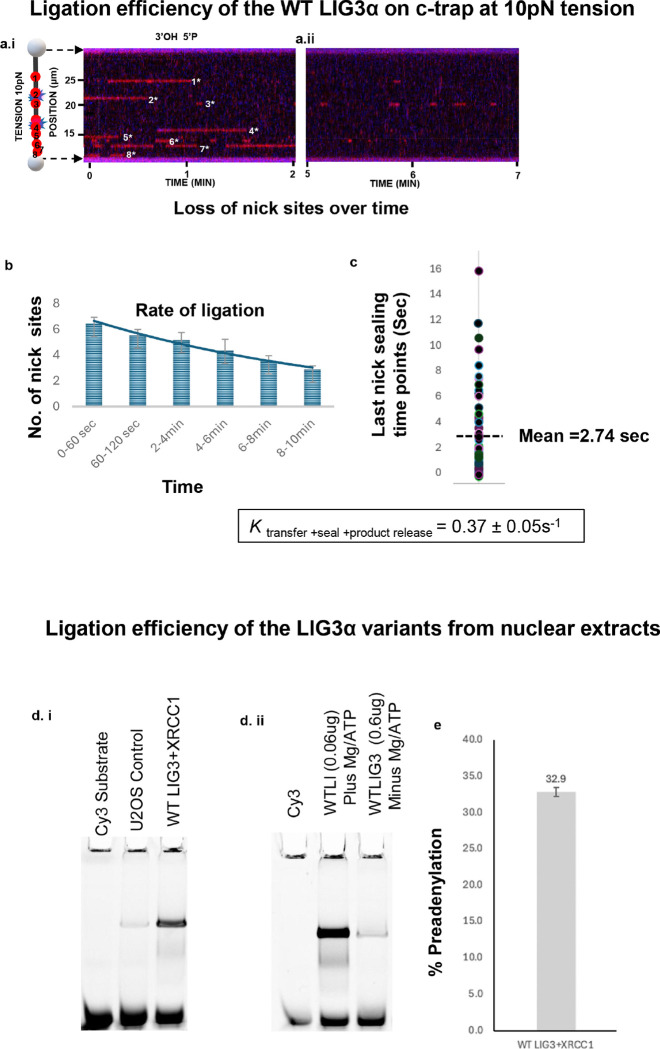
Visualizing nick sealing in real time at 10pN tension and analysis of LIG3α variants showing ligation. **(a)** A corresponding kymograph of an observation for Halo 635-LIG3α-XRCC1-YFP binding to nicked λ DNA digested with nicking enzyme Nt.BspQI generating eight observable ligatable nicks sites at 10 pN tension with an observation window of 2min. The second kymograph is the follow up from the first kymograph showing the last observation window from 5–7 min of data collection. **(b)** Graph showing number of nick sites ligating over time, yielding a rate of ligation. **(c)** Plot of last sealing time points. **(d)** Ligation assay for WTLIG3α used in our nuclear extracts in absence or presence of ATP and Mg^2+^. **(e)** Ligation efficiency based on total adenylation within the nuclear extracts.

LIG3α seals a 3’OH and a 5’-phosphate via a three-step catalytic process that harnesses the energy of ATP. During this process LIG3α catalyzes the transfer of AMP from ATP to the active site lysine 421 that forms an adenylated enzyme intermediate. This intermediate then transfers AMP group to the 5′-phosphate at the break, and 3′-OH attacks the adenylated 5′-phosphate to generate a new phosphodiester bond and release AMP thereby sealing the nick ^2^. While prior bulk biochemical studies have reported the binding kinetics of LIG3α in presence of ATP and Mg^2 + 50^, we found that, under our assay conditions, the addition of Mg^2+^ resulted in infrequent binding for shorter durations (**Fig. S3**). In order to determine how much LIG3α is charged with AMP in the nuclear extracts, we investigated the role of ATP and LIG3α adenylation in two ways. First, we followed the number of LIG3α binding positions, which started with 7–8 binding sites but gradually decreased over time as nicks were ligated. In this way we measured the ligation rate based on lifetime of the last binding event prior to the loss of subsequent binding at that same site. (Fig. 2 ai, aii, b & c). Eventually over a period of 10-min of data collection at 10 pN tension we observed much less frequent nick binding over time (Fig. 2 ai, aii). By taking the mean time of all the last binding events, in which no further binding to those sites were observed, we could calculate an actual ligation rate that is a combination of *k*_AMP transfer_, *k*_seal_, and the rate of product release, which was 0.37 +/− 0.05 s^− 1^ (Fig. 2c). This rate was very close to a previous reported ktransfer, (0.89 s^− 1^), which appears to be the rate-limiting step for ligation ^50^. In the second approach, using bulk biochemical analysis nuclear extracts incubated with nicked DNA duplex in the absence or presence of ATP and Mg^2+^ in our working buffer (see Material and Methods). Under these conditions, the majority of the ligation events were carried out by the tagged LIG3α (Fig. 2di). As expected, there was more ligation under multiple turnover conditions with ATP and Mg^2+^ (Fig. 2dii). By measuring ligation in the absence of ATP and Mg^2+^, we estimate approximately ~ 30% of the LIG3α molecules were charged with AMP during extract preparation (Fig. 2dii, e).

### 3’OH and 5’OH moieties and catalytically dead Halo-LIG3α (K421A), alters nick binding kinetics.

**Figure 3:**
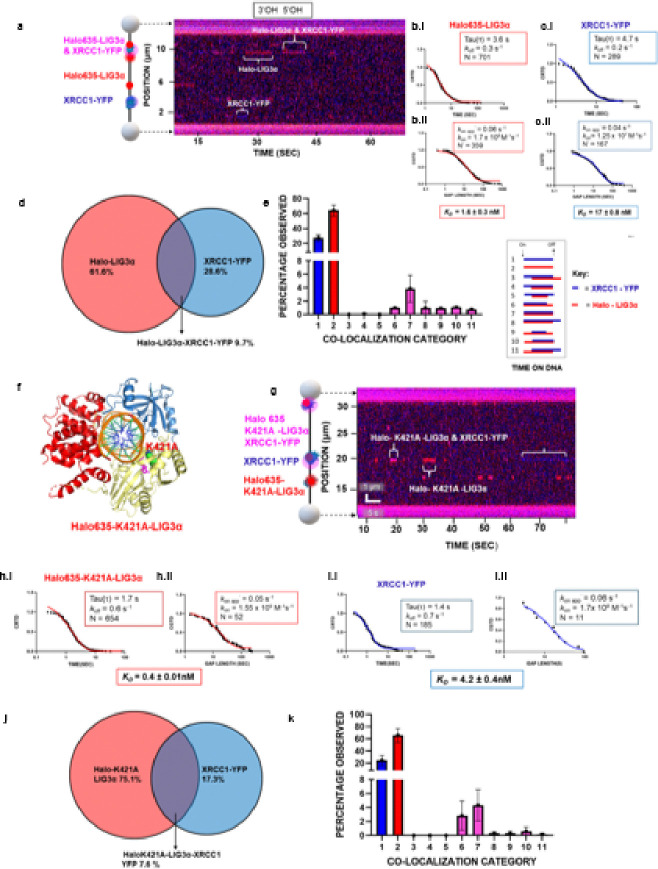
3’OH and 5’OH moieties and catalytically dead Halo-LIG3α (K421A), alters nick binding kinetics. **(a)** A corresponding kymograph of an observation for Halo 635-LIG3α-XRCC1-YFP binding calf intestinal alkaline phosphatase (CIP) treated 10 nicked λ DNA generating 8 observable non-ligatable nick sites at 10 pN tension. **(b)** Cumulative residence time distribution (CRTD) and cumulative gap time distribution (CGTD), analysis for Halo 635-LIG3α binding CIP treated nicked λ DNA, with a single exponential fit shown in red. **(c)** Cumulative residence time distribution (CRTD) and cumulative gap time distribution (CGTD), analysis for XRCC1-YFP binding CIP treated nicked λ DNA, with a single exponential fit shown in blue. **(d)** Percentage of events that were Halo 635-LIG3αalone in red and XRCC1-YFP alone in blue, or colocalized together in the middle as pink. **(e)** The distribution of the 11 categories for Halo 635-LIG3α and XRCC1-YFP binding to CIP treated non-ligatable 10 nicked λDNA. Error bars represent the SEM of two experiments. Inset: 11 colocalization categories.**(f)** A representative structural model of catalytically dead N-terminal tagged Halo635-K421A LIG3α bound to nicked DNA (PDB code 3L2P) **(g)** A corresponding kymograph of an observation for Halo 635-K421A LIG3α-XRCC1-YFP binding to10 nicked λ DNA at 10 pN tension. **(h)** Cumulative residence time distribution (CRTD) and cumulative gap time distribution (CGTD), analysis for Halo 635-K421A-LIG3α, with a single exponential fit shown in red. **(i)** Cumulative residence time distribution (CRTD) and cumulative gap time distribution (CGTD), analysis for XRCC1-YFP, with a single exponential fit shown in blue. **(j)** Percentage of events that were Halo 635-K421A LIG3αalone in red and XRCC1-YFP alone in blue, or colocalized together in the middle as pink. **(k)** The distribution of the 11 categories for Halo 635- K421A LIG3α and XRCC1-YFP binding to ligatable 10 nicked λ DNA. Error bars represent the SEM of two experiments.

Pre-adenylated LIG3α transfers the AMP moiety covalently linked to K421 in the NTase domain ^40^ to the 5’-P terminus of the nick generating a 5’−5’ adenylated DNA intermediate (Ap-DNA) complex ^51^. To test the importance of 5’P in nick binding, we pre-incubated the nicked λ DNA with calf-intestinal alkaline phosphatase (CIP) which removes 5’ phosphate groups leaving non-ligatable 5’OH nick termini (see Materials and Methods). Using our single-molecule approach we observed the binding events for Halo 635-LIG3α-XRCC1-YFP binding to CIP-treated nicked λ DNA generating 8 observable non-ligatable nick sites at 10 pN tension as shown in the corresponding kymograph (Fig. 3a). WT LIG3α had ~ 16-fold reduced binding affinity (*K*_*D*_ = 1.64 ± 0.3 nM, Fig. 3b. ii) for the CIP-treated nicked λ DNA (see Table 1). In addition, the binding of XRCC1 to the CIP-treated nicked λ DNA was also reduced, exhibiting an ~ 11-fold weaker affinity (*K*_*D*_ = 17 ± 0.8 nM) (Fig. 3.c. ii, and Table 1). In accord with their reduced binding affinities and shorter binding lifetimes there was ~ 1.7 fold reduced co-localization complexes (9.7%), when compared to the WT (17.1%) and overall fewer co-localization complexes were formed (Fig. 3d & 3e). This clearly indicates that 5’ P plays a crucial role for the proper nick binding and transfer of AMP. These studies are consistent with bulk biochemical studies that have previously shown the LIG3α had a two-fold reduced binding affinity by removal of the 5’-phosphate ^6,43^.

To further probe the function of adenylated K421 in nick binding, we replaced K421 with an alanine residue (Fig. 3f) and measured the bindings kinetics to ligatable nicks using SMADNE following co-expression with XRCC1-YFP. The binding lifetimes for LIG3α K421A-XRCC1 were 3-fold shorter when compared to similar studies with WT-Halo-635-LIG3α (Fig. 3h & i, see Table 1). The durations were then fit to a one phase exponential CRTD fit, yielding an average lifetime for Halo-635-K421A-LIG3α of 1.7 sec (± 0.02), and 1.4 sec (± 0.02), for XRCC1 (Fig. 3h.i & h.ii). When compared to wild type LIG3α, the catalytically dead K421A mutant exhibits 4-fold weaker binding (*K*_*D*_ = 0.4 ± 0.01 nM), Table 1. The shorter lifetime binding of the K421A mutant resulted in a 2-fold reduction in colocalization with XRCC1 i.e., 7.6% when compared to the WT (Fig. 3j). These data indicate that lysine 421 and/or its adenylation contributes to nick binding.

### The catalytic core (CC) of LIG3α competes with the ZnF domain for nick engagement

**Figure 4:**
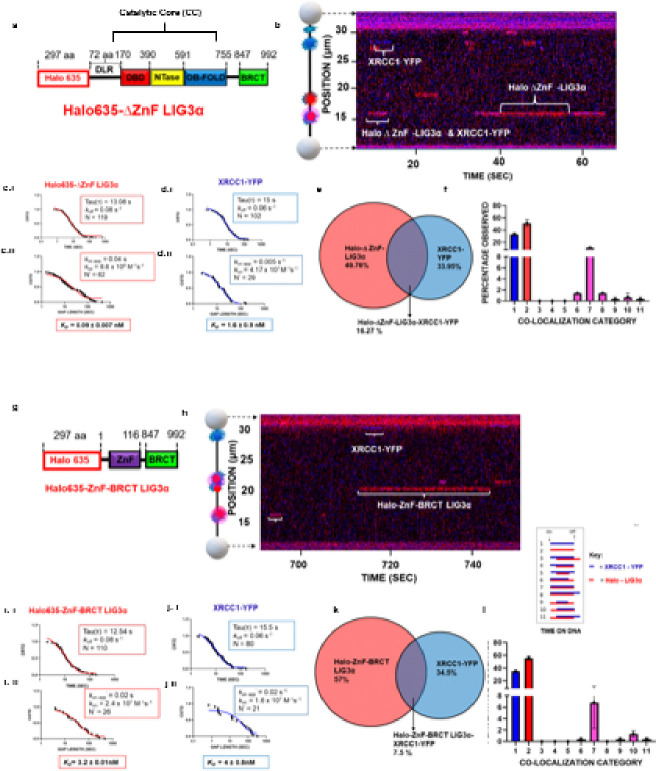
The catalytic core (CC) of LIG3α competes with the ZnF domain for nick engagement **(a)** Domain structure of ΔZnF construct of LIG3α. **(b)** A corresponding kymograph of an observation for Halo 635-ΔZnF LIG3α-XRCC1-YFP binding to λ DNA with 8 observable possible ligatable nick sites at 10 pN tension. **(c)** Cumulative residence time distribution (CRTD) & Cumulative gap time distribution (CGTD), analysis for Halo 635-ΔZnF LIG3α binding nicked λ DNA, with a single exponential fit shown in red. **(d)** Cumulative residence time distribution (CRTD) and cumulative gap time distribution (CGTD), analysis for XRCC1-YFP binding nicked λ DNA, with a single exponential fit shown in blue. **(e)** Percentage of events that were Halo 635-ΔZnF LIG3α alone in red and XRCC1-YFP alone in blue, or colocalized together in the middle as pink. **(f)** The distribution of the 11 categories for Halo 635-ΔZnF LIG3α and XRCC1-YFP binding to nicked λ DNA. Error bars represent the SEM of two experiments. **(g)** A representative domain structural model Halo635 ZnF-BRCT LIG3α. **(h)** A corresponding kymograph of an observation for Halo 635-ZnF-BRCT LIG3α-XRCC1-YFP binding to nicked λ DNA at 10 pN tension. **(i)** Cumulative residence time distribution (CRTD) and cumulative gap time distribution (CGTD), analysis for Halo 635-ZnF-BRCT LIG3α, with a single exponential fit shown in red. **(j)** Cumulative residence time distribution (CRTD) and cumulative gap time distribution (CGTD), analysis for XRCC1-YFP, with a single exponential fit shown in blue. **(k)** Percentage of events that were Halo 635-ZnF-BRCT LIG3α alone in red and XRCC1-YFP alone in blue, or colocalized together in the middle as pink. **(l)** The distribution of the 11 categories for Halo 635-ZnF-BRCT LIG3α and XRCC1-YFP binding to ligatable 10 nicked λ DNA. Error bars represent the SEM of two experiments. Inset: 11 colocalization categories.

While previous bulk biochemical studies with purified proteins have shown that the LIG3α N-terminal zinc finger domain plays an important role in nick binding and ligation under physiological salt concentrations and intermolecular duplex ligation, it is not required for catalysis but instead it appears to function as an initial nick sensor ^43,52–54^. These studies identified that deletion of the ZnF in LIG3α did not significantly change the DNA binding affinity of LIG3α to an intact duplex ^43^. To further understand the role of the LIG3α N-terminal zinc finger ZnF in nick recognition, we expressed versions of LIG3α that either lack the ZnF domain (ΔZnF) or lack the entire catalytic core (LIG3α ZnF-BRCT) and observed their bindings to ligatable nicks (see Fig. 4a, b, g & h). LIG3α ΔZnF binds to ligatable nicked DNA as well as the full-length WT LIG3α construct (*K*_*D*_ = 0.09 ± 0.007 nM versus *K*_*D*_ = 0.1 ± 0.003 nM; Fig. 4c & d, Table 1). In contrast the ZnF-BRCT variant binds with ~ 36-fold weaker affinity than WT LIG3α (*K*_*D*_ = 3.2 ± 0.1 nM) (**Fig. i.i & i.ii**). In the presence of ZnF-BRCT variant, XRCC1 also shows a similar drop in affinity (*K*_*D*_ = 4.0 ± 0.8 nM) (Fig. 4j.i & j.ii). The slower on rates (*k*_*on*_) for both ZnF-BRCT (2.4 ± 0.12 × 10^7^ s^− 1^ M^− 1^) and ΔZnF (8.6 ± 0.6 × 10^8^ s^− 1^ M^− 1^) when compared to WT (1.6 ± 0.04 × 10^9^ s^− 1^ M^− 1^), suggests that nick sensing occurs through a concerted effort of both the ZnF and the catalytic core (CC). Interestingly, there is a two-fold decrease in co-localization of XRCC1 with the ZnF-BRCT variant when compared to the ΔZnF construct, which may indicate that XRCC1 interacts with the CC in addition to the BRCT domain (7.5% versus 16.3%) (Fig. 4k & l). Together these data suggest that there are coordinated rapid handoffs between the ZnF to the CC during nick sensing by WT LIG3α whereas, in the absence of either ZnF or the CC, nick sensing is diminished.

### Robust engagement of LIG3α with undamaged 601 NCP at single molecule level

**Figure 5:**
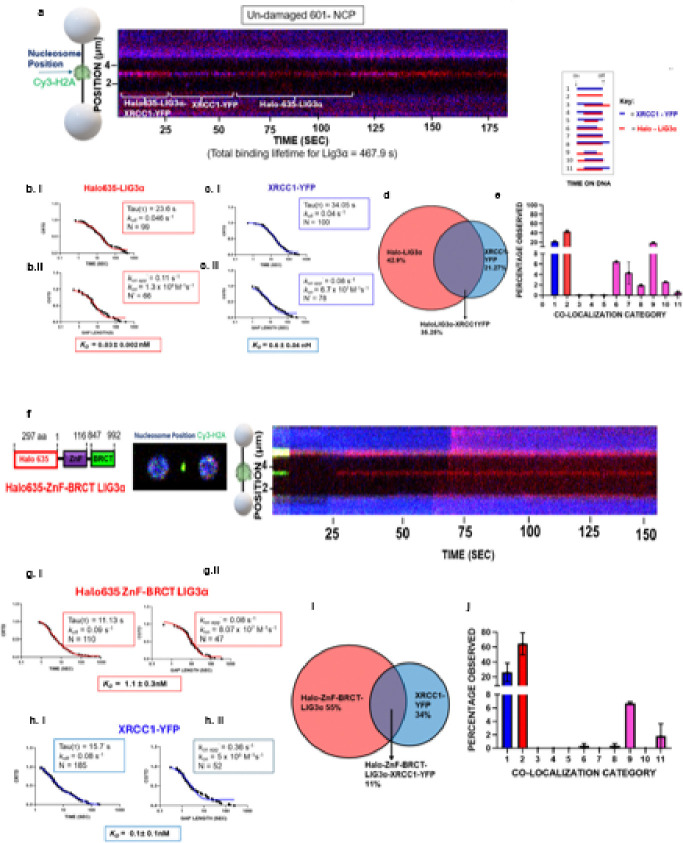
Robust engagement of LIG3α with undamaged 601 NCP at the single molecule level. **(a)** A corresponding kymograph of an observation for Halo 635-LIG3α-XRCC1-YFP binding undamaged 601 NCP at 5 pN tension. **(b)** Cumulative residence time distribution (CRTD) and cumulative gap time distribution (CGTD), analysis for Halo 635-LIG3αbinding undamaged 601 NCP, with a single exponential fit shown in red. **(c)**Cumulative residence time distribution (CRTD) and cumulative gap time distribution (CGTD), analysis for XRCC1-YFP binding, undamaged 601 NCP with a single exponential fit shown in blue. **(d)** Percentage of events that were Halo 635-LIG3α alone in red and XRCC1-YFP alone in blue, or colocalized together in the middle as pink. **(e)** The distribution of the 11 categories for Halo 635-LIG3α and XRCC1-YFP binding to undamaged 601 NCP. Error bars represent the SEM of two experiments. **(f)** A domain model of ZnF-BRCT LIG3α construct, followed by a resultant 2D scan demonstrating colocalization between the Cy3 signal of the nucleosome and the ATTO 647N dye (represented by yellow). A corresponding kymograph of an observation for Halo 635-ZnF-BRCT LIG3α-XRCC1-YFP binding undamaged 601 NCP at 5 pN tension. **(g)** Cumulative residence time distribution (CRTD) and cumulative gap time distribution (CGTD), analysis for Halo 635-ZnF-BRCT LIG3α, with a two-phase exponential fit shown in red. **(h)** Cumulative residence time distribution (CRTD) and cumulative gap time distribution (CGTD), analysis for XRCC1-YFP, with a two-phase exponential fit shown in blue. **(i)** Percentage of events that were Halo 635-ZnF-BRCT LIG3αalone in red and XRCC1-YFP alone in blue, or colocalized together in the middle as pink. **(j)** The distribution of the 11 categories for Halo 635-ZnF-BRCT LIG3α and XRCC1-YFP binding to a nondamaged NCP. Error bars represent the SEM of two experiments.

In the nucleus, DNA repair occurs in the context of chromatin, in particular the nucleosomal core particle ^55^. Previous biochemical studies have examined the association of repair proteins with undamaged NCPs and the activity of BER enzymes on DNA damage within a NCP ^4,56,57^ A drawback with these studies is that the DNA fragments containing NCP have open free DNA ends, which are also bound by LIG3α, as well as other repair proteins and could interfere with LIG3α binding kinetics to nicks ^4,58^. To characterize the interactions of LIG3α and XRCC1 with nucleosomes having no free DNA ends, Cy3-labeled human histone nucleosomes were reconstituted onto a 191 bp DNA duplexes containing a central Widom 601 sequence ^59^. The reconstituted nucleosomes were then ligated into 6 kb biotinylated DNA handles (DNA tethering kit from LUMICKS), and the ligated substrate was tethered between optically trapped streptavidin beads on the C-trap (Fig. 5a & f). Using this approach, we quantified the binding kinetics of LIG3α and XRCC1 to nucleosomes in a substrate that more closely mimics native chromatin. Since DNA tension greater than 5 pN results in unwrapping the distal arm of nucleosomes reconstituted on the 601 sequence, all the experiments with the NCP substrate were performed at 4–5 pN tension to ensure full DNA wrapping ^60^.

Surprisingly, single-molecule imaging revealed that WT-LIG3α avidly binds to an undamaged NCPs with a lifetime of 23.6 ± 0.6 s (Fig. 5b.i) and has a ~ 3.3-fold tighter binding affinity (*K*_*D*_ = 0.03 ± 0.002 nM) compared to a ligatable nick (*K*_*D*_ = 0.1 ± 0.03 nM). However, compared to LIG3α, XRCC1 binds 20-fold less tightly to an undamaged NCP as compared to LIG3α (*K*_*D*_ = 0.6 ± 0.04 nM; Table 2). Order of assembly analysis identified that LIG3α not only avidly binds to the undamaged NCP but also often binds independent of XRCC1 (43%: Fig. 5c). When LIG3α and XRCC1 binding to an NCP overlap, LIG3α almost always binds first prior to XRCC1 engagement (Fig. 5e, see video supplemental Movie 2). To determine the region of LIG3α that mediates NCP binding, we performed single molecule imaging with LIG3α variants either lacking the ZnF or the CC. This domain analysis found that the ZnF-BRCT avidly binds to an undamaged NCP with high affinity (*K*_*D*_ *=* 1.1 ± 0.3nM). This is three-fold tighter than this variant’s binding affinity to a ligatable nick in naked DNA. Notably, deletion of the ZnF binding domain (ΔZnF-LIG3α variant) of LIG3α abrogated the interaction of LIG3α with an undamaged NCP (**Fig. S4**). After identifying the ZnF domain as critical for the NCP interaction, we hypothesized that the R62 & R64 residues within the Znf domain of LIG3α may act as arginine anchors that bind the acidic patch of the nucleosome (**Fig. S4**) ^61^. Indeed, substitution of Arg62 and Arg64 to alanine caused LIG3α to no longer bind to the undamaged NCP (**Fig S4**). Together these data indicate that LIG3α utilizes R62 and R64 of the ZnF domain to presumably bind to the acidic patch of NCPs (see Fig. 7.c).

### Non-ligatable nick position in the NCP alters LIG3α and XRCC1 binding kinetics

**Figure 6:**
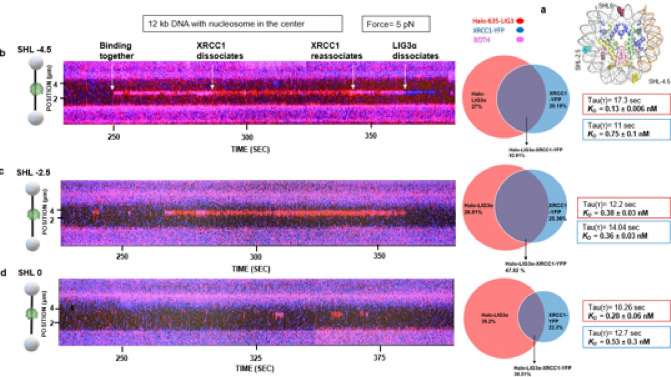
Positioning of non-ligatable nicks within the 601NCP alters LIG3α and XRCC1 binding kinetics. **(a)** Structural model of the nucleosomes generated from PDB 4JJN, with sites of each nick site marked along the structure (with SHL0, SHL-2.5, and SHL-4.5). **(b)** A corresponding kymograph of an observation for Halo 635- LIG3α-XRCC1-YFP binding 601 NCP having the nick at SHL −4.5 position, at 5 pN tension, along with the percentage of co-localized events and binding kinetics (see Table 2). **(c)**A corresponding kymograph of an observation for Halo 635- LIG3α-XRCC1-YFP binding 601 NCP having the nick at SHL −2.5 position, at 5 pN tension, along with the percentage of co-localized events and binding kinetics (see Table 2). **(d)** A corresponding kymograph of an observation for Halo 635- LIG3α-XRCC1-YFP binding 601 NCP having the nick at SHL 0 position, at 5 pN tension, along with the percentage of co-localized events and binding kinetics (see Table 2).

**Figure 7:**
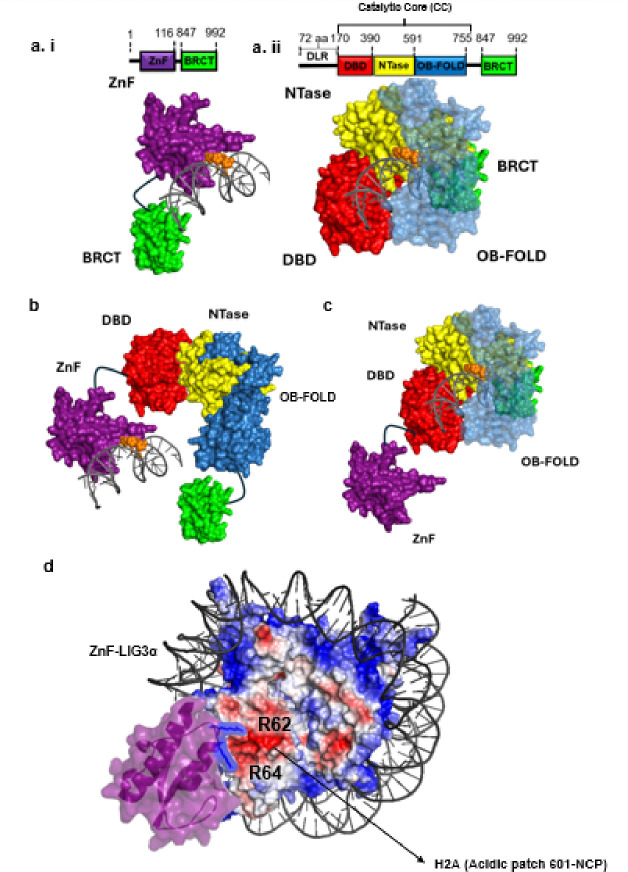
Detection of damage and chromatin by LIG3α. **(a. i)** Model for how the ZnF-BRCT binds to the nicked DNA before it can fully encircle as a jackknife. **(a. ii)**Model of how CC core complex engages to the nick in the absence of the ZnF. The site of the nick is colored orange. **(b. i and b. ii)** Model of full length LIG3a showing dynamic switching between ZnF and CC domains for nick recognition. **(c)** Model showing the Arg anchor R62 and R64 (blue) of the ZnF domain (purple) binding to the acidic patch (red) of LIG3α binding to an undamaged 601-NCP.

Chromatin is subjected to various environmental and enzymological factors that can generate DNA SSBs ^58,62^. Having found that LIG3α binds with high affinity to undamaged NCPs, we examined the binding of LIG3α and XRCC1 to nicks within nucleosomes. Using our 601 NCP substrate, we introduced a non-ligatable nick containing a 3’OH and 5’OH at three different positions within the NCP (one super helical location (SHL) at a time: 0, −2.5, or −4.5). and performed single molecule imaging (Fig. 6a). LIG3α binding lifetimes for SHL − 4.5 were longer (Tau = 17.3 ± 3.2 sec) compared when compared to SHL − 2.5 (Tau = 12.2 ± 3.5 sec) and SHL 0 (Tau = 10.2 sec ± 2.2) but were shorter than binding to the undamaged NCP (Tau = 23.7 ± 0.6 sec). Also, examination of the frequency of co-localization for LIG3α and XRCC1 indicated the highest co-localization of the two proteins occurred when the nicks was a SHL − 4.5, followed by SHL − 2.5, and finally SHL 0 (Fig. 6b, c & d). The CRTD plots fitted a two-phase exponential curve composed of some long binding events (Tau slow), and shorter events (Tau fast) (see **Fig. S5**). We used a two-phase exponential fit assuming that the long binding events (Tau slow), are the ZnF engagements with the nucleosome through the arginine anchor, whereas shorter events (Tau fast) are from the LIG3α ZnF domain disengaging from the nucleosome and showing shorter nick binding. These data suggest a process in which that the ZnF domain of LIG3α can switch from relatively stable binding to the NCP (Tau slow) to rapidly sensing the nicks (Tau fast) embedded within the NCP. While the three SHL nick positions exhibited relatively similar binding kinetics for LIG3α and XRCC1, the highest affinity substrate was SHL − 4.5 (see Table 2). We also examined LIG3α binding kinetics to a single non-ligatable nick in the absence of an NCP, and LIG3α exhibited a decreased binding affinity (*K*_*D*_ = 2.5 ± 0.1 nM), similar to the CIP-treated nicked λ DNA substrate (*K*_*D*_ = 1.6 ± 0.3 nM) (**Fig. S6**). These data indicate that the nick sensing role of ZnF causes release from binding to the nucleosome, causing a decrease in the overall dwell time of LIG3a on the nick containing nucleosomes, with the nick at SHL 0 being the strongest competitor for nucleosome binding (Fig. 7).

## Discussion

DNA nick sealing is an essential step in the SSB and BER pathways orchestrated by XRCC1 and LIG3α ^40,63,64^. This single molecule study provides new mechanistic insights into the kinetics of LIG3α and XRCC1 interactions with substrates containing non-ligatable and ligatable SSBs in naked DNA, nucleosomes without DNA damage and nucleosomes with non-ligatable nicks. Since the DNA substrates were embedded in long DNA tethers suspended between two beads, this single molecule approach overcomes limitations of other biochemical and biophysical approaches in which these nicked DNA substrates have free DNA ends. We discovered that LIG3α has a 15-fold higher affinity for ligatable nicks (*K*_*D*_ = 0.10 nM) than XRCC1 (*K*_*D*_ = 1.5 nM), and that the two proteins are not obligate heterodimers, with co-localizing events at nicks constituting only 17.1% of the total events. Since the steady state levels of LIG3α are reduced in cells expressing mutant versions XRCC1 are that are defective in complex formation with LIG3α, it has been assumed that XRCC1 is required for the stability of LIG3α and that these proteins functions as a heterodimer in nuclear DNA repair. While the low protein concentrations of the nuclear extract used for the single molecule studies may result in the dissociation of the LIG3α-XRCC1 complex, it is possible that the function of XRCC1 is to interact with LIG3α in the cytoplasm and direct its transport into the nucleus where it is protected from proteolysis and can function independently from XRCC1. Notably, XRCC1 is not present in mitochondria and the levels and function of mitochondrial LIG3α, which has an N-terminal mitochondrial targeting sequence generated by alternative translation initiation, are not impacted in *xrcc1* cells with reduced total cellular levels of LIG3α ^17^.

Previous studies using bulk fluorescence polarization assays reported a *K*_*D*_ for LIG3α binding to nicks of 300 nM, three orders of magnitude less tight than what we determined ^65^. One confounder in those studies was that the nick was in a short duplex and, since LIG3α has affinity for the ends of duplex DNA, the authors were forced to work at 250 mM NaCl to reveal specific binding to nicks. By embedding nicks in a long DNA substrate and measuring on and off rates we have overcome this previous limitation. Recent bulk biochemistry SPR experiment indicate XRCC1 has affinity (54 nM) for nicks which is considerably lower affinity than what we measured in our studies (K_D_ = 0.6 nM) ^66^. Our real-time tracking of order assembly (Fig. 1) indicates that when the proteins colocalize at nicks either LIG3α and XRCC1 bind together at the site of the nick or LIG3α binds first recruiting XRCC1 through the BRCT domain interaction. We observed ligation in real time at the single molecule level, with the rate of apparent rate of nick ligation for LIG3α was 0.37 +/− 0.05 sec^− 1^ (Fig. 2). Previous bulk biochemical single turnover experiments indicated that while the rate of sealing is rather fast, 19 sec^− 1^, and the AMP transfer step from LIG3α to the 5’phosphate was rate limiting at 0.89 sec^− 1^ at 37°C ^50^. While our data were generated at 25°C and in a different buffer, these two values agree very well. To understand the effect of adenylation in LIG3α, we tested a K421A variant which is catalytical dead due to the inability to be charged with AMP. This variant had a three-fold lower *K*_*D*_ for nicks due to its shorter dwell time. We determined that ~ 30% of overexpressed LIG3α is fully charged with AMP (**Fig. S3**), thus some of the shorter binding events we are observing with WT protein, could also be due to uncharged LIG3α. We sought to examine the effect of ATP and Mg^2+^ in the extract and reaction buffer, but this was unsuccessful since Mg^2+^ seemed to greatly decrease binding to nicks in our system (**Fig. S3**).

Domain mapping studies provided new insights into how LIG3α detects and processed ligatable nicked DNA. Removal of the ZnF domain, caused 2.5-fold longer dwell times (see Table 1). The on rates were 5-fold more for ΔZnF LIG3α construct (8.6 ± 0.6 × 10^8^ s^− 1^ M^− 1^) when compared to full length construct (1.66 ± 0.04 × 10^9^ s^− 1^ M^− 1^) and a *KD* within error of the full-length protein (see Table 1). Surprisingly the ZnF-BRCT construct of LIG3α also exhibited a two-fold longer dwell time compared to full length (see Table 1) but had a 1.4-fold longer on rate (1.66 ± 0.04 × 10^9^ s^− 1^ M^− 1^) when compared to the full-length protein (1.66 ± 0.04 × 10^9^ s^− 1^ M^− 1^). These data clearly suggest that the ZnF and the CC domain effectively compete for nick binding in the full-length protein in partial support of the jack-knife model first proposed by Ellenberger and coworkers ^43^. Rapid shuttling between the ZnF and the CC increases the binding affinity of full length LIG3α for nicks. These data (Fig. 4) are consistent with previous biochemical studies showing that the ZnF domain is required for efficient ligation of SSB ^67 68^ and also blunt DNA ends ^6^. However, since the ZnF-BRCT construct bound 32-fold less tight than the full length, it would seem that the ZnF of LIG3α provides another important function to LIG3α, as it is the only mammalian DNA ligase containing a ZnF.

This work presented in Fig. 5 unequivocally established that LIG3α binds to undamaged NCPs with high affinity (*K*_*D*_ = 0.03 nM). Subsequent domain analysis indicated that two critical Arg in the ZnF domain are essential for the high affinity binding to a nucleosome. Loss of the ZnF or substituting both Arg62 and Arg64 for Ala in LIG3α completely abrogated binding to nucleosomes, whereas the ligase activity of these variants was higher than WT (**Fig. S3h & i**). It remains unknown why the ZnF motif evolved in some species, but our discovery that the arginine anchor resides within the ZnF and that the RARA sequence in the arginine anchor is highly conserved in all organisms containing a ZnF on LIG3α (see supplemental **Fig. S4**) argues that this motif has a dual role in chromatin architecture and DNA repair within nucleosomes compared to other ligases that lack the ZnF domain or arginine anchor ^1^. In accord with our results, previous mass spec screening experiments of proteins that bound to different regions of nucleosomes found that LIG3a bound to nucleosome but did not identify a precise binding position within the nucleosome, probably due to the confounding binding to the free double-strand ends in this study ^4^. Notably, the structurally conserved ZnF1 in PARP1 does not contain these arginine anchors, suggesting that PARP1 potentially binds nucleosomes through an alternate interaction that does not compete with LIG3α. Arginine anchors have been discovered in a number of proteins that bind directly to nucleosomes, but never to our knowledge in a ZnF domain of a protein ^61^. We also found that placing a non-ligatable nick in a nucleosome apparently weakens the overall affinity of LIG3α to a nucleosome (Table 2), mostly through a decrease in binding lifetimes. These data suggest that the binding affinity of the ZnF to the acidic patch is altered by nearby nicks, implying that in a biological setting LIG3α may be “pre-positioned” on chromatin for immediate response to sealing nicks. It should be noted that modeling of LIG3α CC domain with nicks within the nucleosomes at SHL 0, −2.5, and − 4.5 would indicate it cannot engage nicks fully without significant nucleosome unwrapping and the transient binding is probably due only to the ZnF engagement with the nick (**Fig. S5**). This is consistent with the finding that nicks in nucleosomes are ligated more slowly ^56^.

The relatively high affinity for NCPs without DNA breaks points to roles of LIG3α outside of DNA repair and may be more relevant to its role in chromatin structure and remodeling during transcription and DNA replication. LIG3α binding to histones within a nucleosome may regulate chromatin state and play an important role in the chromatin architecture. Early evidence has suggested that LIG3α has an affinity for condensed chromatin in metaphase chromosomes ^47^. Other factors such as post-translational modifications (PTMs) may alter interactions of LIG3α with chromatin. To this end it is interesting to note that LIG3α is phosphorylated on Ser123 during early S phase by CDK2 ^69^ and that oxidative stress induces loss of Ser123 phosphorylation in an ATM dependent manner probably through activation of a phosphatase. Also, a recent study suggests that LIG3α can be recruited into condensates of PARylated nucleosomes. Thus, histone modifications and PARP1 activity may play an important role in LIG3α binding to chromatin ^70^. Future single molecule studies using nucleosome arrays and other protein factors, such as PARP1 will provide insights into how LIG3α interacts with chromatin and is shuttled towards sites of damage during DNA replication, repair, and transcription.

## Materials And Methods

### Lead Contact and Material Availability

Further information and requests for resources and reagents should be directed to and will be fulfilled by the Lead Contact, Bennett Van Houten (vanhoutenb@upmc.edu).

### Method Details

#### Cell lines

U2OS cells were cultured in 5% oxygen in Dulbecco’s Modified Eagle Medium (DMEM) supplemented with 4.5g/l glucose, 10% fetal bovine serum (Gibco), 5% penicillin/streptavidin (Life Technologies). To obtain transient overexpression of the fluorescent-tagged proteins of interest, 4 ug of plasmid per 4 million cells was used to transfect using the lipofectamine 2000 reagent and protocol, including 4–6 h of lipofectamine treatment before changing media and letting the plasmids express overnight (Thermo Fisher Cat# L3000008). Cells with overexpressed HaloTag fusions were treated with 100 nM (~10–100-fold molar excess) of fluorescent HaloTag ligand for 30 minutes at 37° C (Janelia Fluor^®^ 635 or 552 HaloTag^®^ Ligand from Dr. Luke Lavis Laboratory, Janelia Research Campus).

#### Nuclear extraction

Nuclear extraction was performed the day after transient transfection using a nuclear extraction kit from Abcam (ab113474) as in the previously reported single-molecule method (Schaich et al., 2023). Resultant nuclear extracts were aliquoted and flash-frozen in liquid nitrogen prior to storage at −80 C in single-use aliquots. Upon use for single-molecule experiments, nuclear extracts were immediately diluted after thawing in buffer for experiments at typically a ratio of 1:10. Concentrations of labeled proteins were determined by comparing the background signal to standard curves of known concentrations, and the efficiency of the HaloTag labeling reaction as well as presence of free HaloTag dye was monitored vis SDS- PAGE (**Fig. S4**).

#### Western blots of overexpressed proteins from nuclear extracts

Various amounts of extracts and purified proteins were loaded onto 4–20% tris-glycine polyacrylamide gels (Invitrogen; XP04202BOX) (**Fig. S1**). Transfer was performed using polyvinylidene difluoride membrane followed by blocking in 20% nonfat dry milk (diluted in PBST: phosphate-buffered saline containing 0.1% Tween 20) for 1 h at room temperature. Membranes were incubated with primary antibodies for 2 h at room temperature or overnight at 4 °C, washed 3 × 10 min in PSBT, and incubated with peroxidase conjugated secondary antibodies for 1 h at room temperature. Membranes were washed again before developing using SuperSignal West Femto Maximum Sensitivity Substrate (Thermo Fisher Scientific; #34095). Primary antibodies used: LIG3α (1:1000; GTX #GTX103172), Secondary antibodies used: anti-rabbit IgG (1:50,000),). Primary antibodies used: XRCC1 (1:1000; abcam #ab134056), Secondary antibodies used: anti-rabbit IgG (1:50,000)

#### Nucleosome reconstitution

Histones were reconstituted onto the 601-sequence using a previously described salt dialysis protocol (Ryan et al., 2023; Schnable et al., 2024). Briefly, human histone H3 C96S C110A, H2A K119C, H2B, and H4 (Histone Source at Colorado State University) were incubated with either H2A/H2B or H3/H4 in 2 mg/mL guanidinium buffer at room temperature for 2 hours, dialyzed against high salt refolding buffer a total of 3 times, and at least 8 hours for each exchange at 4°C. H2A K119C/H2B dimers and H3 C96S C110A/H4 tetramers were purified using a Superdex 200 column. To perform the maleimide labeling, 2fold molar excess Cy3- malemidie dye was added H2A K119C/H2B dimer in 0.7mM TCEP. Cy3-maleimide dye was added to the H2A K119C in a 2:1 molar ratio and incubated at room temperature for 1 hour while rocking. Reactions was quenched with 10 mM DTT, the dimer was purified over a Superdex S200 column, and frozen in 50% glycerol. After confirming the stoichiometry with SDS gel, add equal volume of 100% glycerol to store the H2A K119C/H2B dimer and the H3 C96S C110A/H4 tetramer at −20°C. To reconstitute an undamaged nucleosome core particle on DNA, the following ultramer sequences were ordered from IDT:

**Top Strand: 5’-phosphate**-CAAC TGA GAC CAT GTA CCC AGT TCG AAT CGG ATG TAT ATA TCT GAC ACG TGC CTG GAG ACT AGG GAG TAA TCC CCT TGG CGG TTA AAA CGC GGG GGA CAG CGC GTA CGT GCG TTT AAG CGG TGC TAG AGC TGT CTA CGA CCA ATT GAG CGG CCT CGG CAC CGG GAT TCT CGA TAA CTC AGC AAT AGT GGG TCT CA– 3’

**Bottom strand: 5’- phosphate** -ACCA TGA GAC CCA CTA TTG CTG AGT TAT CGA GAA TCC CGG TGC CGA GGC CGC TCA ATT GGT CGT AGA CAG CTC TAG CAC CGC TTA AAC GCA CGT ACG CGC TGT CCC CCG CGT TTT AAC CGC CAA GGG GAT TAC TCC CTA GTC TCC AGG CAC GTG TCA GAT ATA TAC ATC CGA TTC GAA CTG GGT ACA TGG TCT CA – 3’

To generate NCPs with single-strand breaks, the “Top Strand” oligonucleotide was annealed to sets of two complementary oligonucleotides to generate non-ligatable nicks in defined locations as follows:

##### For SHL0:

**Nick_SHL0_Istrand1:** 5’-phosphate- AC CAT GAG ACC CAC TAT TGC TGA GTT ATC GAG AAT CCC GGT GCC GAG GCC GCT CAA TTG GTC GTA GAC AGC TCT AGC ACC GCT TAA ACG CAC GTA CGC G

**Nick_SHL0_Istrand2**: CTG TCC CCC GCG TTT TAA CCG CCA AGG GGA TTA CTC CCT AGT CTC CAG GCA CGT GTC AGA TAT ATA CAT CCG ATT CGA ACT GGG TAC ATG GTC TCA

##### For SHL-2.5:

**Nick_2.5_Istrand1** (IDT Ultramer): /5Phos/AC CAT GAG ACC CAC TAT TGC TGA GTT ATC GAG AAT CCC GGT GCC GAG GCC GCT CAA TTG GTC GTA GAC AGC TCT AGC ACC GCT TAA ACG CAC GTA CGC GCT GTC CCC CGC GTT TTA ACC GC

**Nick_2.5_Istrand2**: CAA GGG GAT TAC TCC CTA GTC TCC AGG CAC GTG TCA GAT ATA TAC ATC CGA TTC GAA CTG GGT ACA TGG TCT CA

##### For SHL-4.5:

**Nick_4.5_Istrand1 (IDT Ultramer):** /5Phos/AC CAT GAG ACC CAC TAT TGC TGA GTT ATC GAG AAT CCC GGT GCC GAG GCC GCT CAA TTG GTC GTA GAC AGC TCT AGC ACC GCT TAA ACG CAC GTA CGC GCT GTC CCC CGC GTT TTA ACC GCC AAG GGG ATT ACT CCC TAG TCT

**Nick_4.5_Istrand2**: CCA GGC ACG TGT CAG ATA TAT ACA TCC GAT TCG AAC TGG GTA CAT GGT CTC A

The annealed DNA, H2A K119C/H2B dimer, and the H3 C96S C110A/H4 tetramer are mixed in a 1:2:1 molar ratio and equilibrate in dialysis tubing against high salt buffer for 30 minutes. The high salt was then removed via dialysis, the reconstituted nucleosome concentrated, and heat shocked at 55°C for 30 minutes. Then, sub-nucleosomes and free DNA were removed using a 10–40% sucrose gradient for 40 hours at 125,000 ×G at 4°C. Fractions containing reconstituted nucleosomes are combined, buffered exchanged into TE buffer, and concentrated to ~1 μM andstored at 4°C. Native PAGE analysis (**Fig. S7**) revealed that these octamers were stable in these conditions for at least 3 months.

#### DNA substrate generation

Lambda DNA was biotinylated and isolated for C-trap experiments as previously described, with aliquots stored at 20 ng/μL at −20° C (Schaich et al., 2023). After thawing aliquots, they were stored at 4° C for up to 2 weeks and then discarded. DNA with single-stranded breaks (nicked DNA) was generated by digesting 1 ug of DNA with the nickase Nt.BspQI (NEB) to generate 10 nicks (8 observable), cutting on the 3’ side of its recognition sequence. Of note, this nickase cuts outside of its recognition sequence, so each nick is flanked by unique DNA sequences, and binding events on this DNA represent an average of all the sequences. Two of these substrates are not observable because one is too close to the streptavidin beads, and two others are so close that they cannot be optically differentiated. After nicking, substrates were aliquoted and stored at −80° C for up to one year ^49^.

To tether defined substrates like the nucleosomes or substrates with a single ligatable nick, the “DNA tethering kit” from Lumicks was utilized. The protocol was followed as per the manufacturer’s instructions: briefly 50 pmol of the DNA fragment was incubated with the two biotinylated 6 kb handles, ligase, and ligase buffer, and placed in the dark to ligate overnight at room temperature. Following ligation, samples were diluted 1:300, and Cy3 fluorescence utilized to confirm tethered samples had labeled NCPs before data collection.

#### Single-molecule experiments

##### DNA tether formation and positioning

Single-molecule experiments were performed on a LUMICKS C-Trap instrument (Hashemi Shabestari et al., 2017). For experiments with lambda DNA (~40 kb), channels one, two, and three were filled with 4.38 μm polystyrene streptavidin beads (LUMICKS), biotinylated DNA, and buffer of interest, respectively, with trapping lasers set to 100%, 30% overall power, and 50% Trap 1 split. For the 12 kb substrates, trapping laser power was reduced to 15% overall power, and 1.5–1.7 μm streptavidin beads were utilized. All three channels were flowed at a pressure of 0.3 bar to maintain laminar flow while catching beads in each trap. Then the traps were moved to channel two and distance varied between the traps while looking for a force response to tether a DNA substrate between the two beads as expected by the extensible Worm-like Chain Model ^71^.Channel three and four (containing the nuclear extracts of interest) were flowed at 0.3 bar for at least 10 s to introduce nuclear extracts into the flow cell while keeping the DNA substrate in the buffer alone. DNA tension was then defined in the absence of laminar flow (either 4–5 pN for wrapped NCPs or 10 pN for dsDNA or unwrapped DNA) and then kymographs were collected when the DNA moved to the nuclear extract.

##### Confocal imaging

YFP was excited with a 488 nm laser and emission collected in a 500–550 nm band pass filter, and HaloTag-JF-635 was excited with a 638 nm laser and emission collected in a 650–750 nm band pass filter. All data was collected with a 1.2 NA 60X water emersion objective and fluorescence measured with single-photon avalanche photodiode detectors. Each laser was set to 5% power and scanned at a rate of 10 frames per second. This framerate allowed for a pulsed excitation approach and a ~threefold increase in fluorophore lifetime before photobleaching compared to continuous scanning ^49^.

#### Data analysis

Single molecule data was exported with Bluelake and analyzed using custom software from LUMICKS (Pylake). The utility C-Trap .h5 Visualization GUI was used for figure generation (Watters, 2020). KymoWidgetGreedy widget from LUMICKS was utilized for line tracking of single-molecule events, performing a gaussian fit over the fluorescent intensity (Mangeol et al., 2016). After tracking the lines, the position and time data for each line was used to determine each line’s duration and the duration of gaps between lines for on rate calculation. As previously reported, YFP blinking was observed to last up to 2 seconds (Dickson et al., 1997), thus events that occurred at the same position less than two seconds apart were connected and considered one binding approach as blinking ^49^. Python scripts used to calculate photobleaching decay constants and colocalization analysis have been deposited at https://harbor.lumicks.com/scripts.

#### Photobleaching analysis

Photobleaching decay constants were determined as previously described for each fluorophore by collecting kymographs with continuous exposure on fluorophores nonspecifically adsorbed at the bottom of the slide. Total intensities were binned by one second intervals and fit to an exponential decay function. To examine the impact of photobleaching on the measured off rates, both the raw values and corrected lifetimes for each dataset are shown in Table S1. As the correction caused only slightly altered most values, the raw values are reported in the text.

### Quantification and Statistical Analysis

For each experiment, the number of observations analyzed has been included in the figure and/or in the figure legends. The types of errors displayed are also indicated in the figure legends and tables. Each dataset represents at least two replicates with two batches of nuclear extracts, and the datasets with nucleosomes combine two batches of reconstitution for each type (undamaged and each nick position).

### Biochemical

#### Assays Buffers:

Buffer used for Ligation Efficiency with Nuclear Extract:

30mM Tris-HCl (pH 7.8), 10mM DTT, 10mM MgCl_2_, 1mM ATP and ddsH2O.

Buffer used for ligation efficiency without Mg/ATP: 30mM Tris-HCl (pH 7.8) and 10mM DTT in ddsH2O.

#### Antibodies for WB:

First antibody: LIG3 (GeneTex Cat#103172) rabbit, 1:2000, 1h, RT

Secondary antibody: HRP anti rabbit (BIO RAD Cat# 1706515) 1:20000, 45min, RT

#### Oligos used:

Upper left 5’ Cy3: Cy3/CGA CGG CCA GTG CAG GGT TTC

Upper right 5’ phosphorylated: GTA AAG TCA CGA CCG TCA TGC

Lower template biotinylated: biotin/GCA TGA CGG TCG TGA CTT TAC GAA ACC CTG CAC TGG CCG TCG

#### Annealing of the oligos:

Mix all the 3 oligo strands together in equal molar amounts. Heat the mixed oligonucleotides to 94°C and gradually cool 1.5°C for 1 minutes each step.

#### Ligation Assay:

Ligation assay was conducted with a Cy3 labeled nick duplex containing biotin on the full-length strand in 10 μL of buffer (30 mM Tris-HCl, pH 7.8, 10 mM DTT, 10 mM MgCl_2_, and 1 mM ATP) containing 3 μL of nuclear extract for 10 minutes at room temperature.

Streptavidin magnetic beads (washed three times in 30 mM Tris-HCl (pH 7.5) were reconstituted 1x ligation buffer. The biotinylated oligos were captured by the addition of 30 μL of streptavidin magnetic beads to each tube and incubate for 10 minutes at room temperature and the beads captured by magnetic pulldown to remove unbound oligos.

Stop buffer, 20 μL (formamide, 30 mM EDTA, and 1.5% SDS) was added to the magnetic beads, and denatured for 5 minutes at 100°C. After another magnetic pulldown to separate the streptavidin beads from the annealed the reaction (20 μL) was analyzed by electrophoresis in a 20% PAGE urea sequencing gel and ran at 250 volts.

#### Western Blotting:

The indicated amount of soluble fraction and purified Lig3+XRCC1 were loaded onto a 7.5% SDS-PAGE gel, which was run at 100 volts until the dye front ran off the gel.

The gel was transferred to a PVDF membrane at 100 volts for 2 hours. The membrane was blocked overnight at 4°C with 5% bovine serum albumin (BSA) in 1x Tris buffer (10 mM, pH 7.5). The membrane was then incubated with a primary antibody against LIG3 for 1 hour at room temperature. The membrane was washed 3 times for 5 minutes each and incubated with an HRP-conjugated anti-rabbit antibody for 45 minutes at room temperature. Finally, the membrane was washed three times for ten minutes each before detection.

## References

[1] Tomkinson A. E., Vijayakumar S., Pascal J. M. & Ellenberger T. DNA ligases: structure, reaction mechanism, and function. Chem Rev 106, 687–699 (2006). 10.1021/cr040498d 16464020

[2] Ellenberger T. & Tomkinson A. E. Eukaryotic DNA ligases: structural and functional insights. Annual review of biochemistry 77, 313–338 (2008). 10.1146/annurev.biochem.77.061306.123941 PMC2933818 18518823

[3] Martin I. V. & MacNeill S. A. ATP-dependent DNA ligases. Genome Biol 3, Reviews3005 (2002). 10.1186/gb-2002-3-4-reviews3005 11983065 PMC139351

[4] Skrajna A. Comprehensive nucleosome interactome screen establishes fundamental principles of nucleosome binding. Nucleic Acids Res 48, 9415–9432 (2020). 10.1093/nar/gkaa544 32658293 PMC7515726

[5] Sallmyr A., Bhandari S. K., Naila T. & Tomkinson A. E. Mammalian DNA ligases; roles in maintaining genome integrity. J Mol Biol, 168276 (2023). 10.1016/j.jmb.2023.168276 37714297 PMC10843057

[6] Cotner-Gohara E. Human DNA ligase III recognizes DNA ends by dynamic switching between two DNA-bound states. Biochemistry 49, 6165–6176 (2010). 10.1021/bi100503w 20518483 PMC2922849

[7] Kaminski A. M. Structures of DNA-bound human ligase IV catalytic core reveal insights into substrate binding and catalysis. Nat Commun 9, 2642 (2018). 10.1038/s41467-018-05024-8 29980672 PMC6035275

[8] Pascal J. M., O’Brien P. J., Tomkinson A. E. & Ellenberger T. Human DNA ligase I completely encircles and partially unwinds nicked DNA. Nature 432, 473–478 (2004). 15565146 10.1038/nature03082

[9] Gao Y. DNA ligase III is critical for mtDNA integrity but not Xrcc1-mediated nuclear DNA repair. Nature 471, 240–244 (2011). 10.1038/nature09773 21390131 PMC3079429

[10] Lakshmipathy U. & Campbell C. The human DNA ligase III gene encodes nuclear and mitochondrial proteins. Mol Cell Biol 19, 3869–3876 (1999). 10207110 10.1128/mcb.19.5.3869 PMC84244

[11] Simsek D. Crucial role for DNA ligase III in mitochondria but not in Xrcc1-dependent repair. Nature 471, 245–248 (2011). 10.1038/nature09794 21390132 PMC3261757

[12] Dulic A. BRCT domain interactions in the heterodimeric DNA repair protein XRCC1-DNA ligase III. Biochemistry 40, 5906–5913 (2001). 11352725 10.1021/bi002701e

[13] Nash R. A., Caldecott K. W., Barnes D. E. & Lindahl T. XRCC1 protein interacts with one of two distinct forms of DNA ligase III. Biochemistry 36, 5207–5211 (1997). 9136882 10.1021/bi962281m

[14] Cuneo M. J., Gabel S. A., Krahn J. M., Ricker M. A. & London R. E. The structural basis for partitioning of the XRCC1/DNA ligase III-{alpha} BRCT-mediated dimer complexes. Nucleic Acids Res 39, 7816–7827 (2011). 10.1093/nar/gkr419 21652643 PMC3177190

[15] Hammel M. An atypical BRCT-BRCT interaction with the XRCC1 scaffold protein compacts human DNA Ligase IIIalpha within a flexible DNA repair complex. Nucleic Acids Res 49, 306–321 (2021). 10.1093/nar/gkaa1188 33330937 PMC7797052

[16] Kirby T. W. Nuclear Localization of the DNA Repair Scaffold XRCC1: Uncovering the Functional Role of a Bipartite NLS. Sci Rep 5, 13405 (2015). 10.1038/srep13405 26304019 PMC4548243

[17] Lakshmipathy U. & Campbell C. Mitochondrial DNA ligase III function is independent of Xrcc1. Nucleic Acids Res 28, 3880–3886 (2000). 10.1093/nar/28.20.3880 11024166 PMC110795

[18] Lakshmipathy U. & Campbell C. Antisense-mediated decrease in DNA ligase III expression results in reduced mitochondrial DNA integrity. Nucleic Acids Res 29, 668–676 (2001). 11160888 10.1093/nar/29.3.668 PMC30390

[19] Demin A. A. XRCC1 prevents toxic PARP1 trapping during DNA base excision repair. Mol Cell 81, 3018–3030.e3015 (2021). 10.1016/j.molcel.2021.05.009 34102106 PMC8294329

[20] Demin A. A. XRCC1 prevents toxic PARP1 trapping during DNA base excision repair. Molecular Cell 81, 3018–3030.e3015 (2021). 10.1016/j.molcel.2021.05.009 34102106 PMC8294329

[21] Fan J. & Wilson D. M. 3rd. Protein-protein interactions and posttranslational modifications in mammalian base excision repair. Free Radic Biol Med 38, 1121–1138 (2005). 10.1016/j.freeradbiomed.2005.01.012 15808410

[22] Ahel I. The neurodegenerative disease protein aprataxin resolves abortive DNA ligation intermediates. Nature 443, 713–716 (2006). 10.1038/nature05164 16964241

[23] Wang H. Mutant FUS causes DNA ligation defects to inhibit oxidative damage repair in Amyotrophic Lateral Sclerosis. Nat Commun 9, 3683 (2018). 10.1038/s41467-018-06111-6 30206235 PMC6134028

[24] Hoch N. C. XRCC1 mutation is associated with PARP1 hyperactivation and cerebellar ataxia. Nature 541, 87–91 (2017). 10.1038/nature20790 28002403 PMC5218588

[25] Wang H. C. DNA ligase III as a candidate component of backup pathways of nonhomologous end joining. Cancer Res 65, 4020–4030 (2005). 10.1158/0008-5472.Can-04-3055 15899791

[26] Sallmyr A. & Tomkinson A. E. Repair of DNA double-strand breaks by mammalian alternative end-joining pathways. J Biol Chem 293, 10536–10546 (2018). 10.1074/jbc.TM117.000375 29530982 PMC6036210

[27] Simsek D. DNA ligase III promotes alternative nonhomologous end-joining during chromosomal translocation formation. PLoS genetics 7, e1002080 (2011). 10.1371/journal.pgen.1002080 21655080 PMC3107202

[28] Simsek D. & Jasin M. Alternative end-joining is suppressed by the canonical NHEJ component Xrcc4-ligase IV during chromosomal translocation formation. Nature structural & molecular biology 17, 410–416 (2010). 10.1038/nsmb.1773 PMC3893185 20208544

[29] Arakawa H. & Iliakis G. Alternative Okazaki Fragment Ligation Pathway by DNA Ligase III. Genes (Basel) 6, 385–398 (2015). 10.3390/genes6020385 26110316 PMC4488670

[30] Han L., Masani S., Hsieh C. L. & Yu K. DNA ligase I is not essential for Mammalian cell viability. Cell reports 7, 316–320 (2014). 10.1016/j.celrep.2014.03.024 24726358 PMC4593317

[31] Le Chalony C. Partial complementation of a DNA ligase I deficiency by DNA ligase III and its impact on cell survival and telomere stability in mammalian cells. Cellular and molecular life sciences : CMLS 69, 2933–2949 (2012). 10.1007/s00018-012-0975-8 22460582 PMC3417097

[32] Bhandari S. K. Unchanged PCNA and DNMT1 dynamics during replication in DNA ligase I-deficient cells but abnormal chromatin levels of non-replicative histone H1. Sci Rep 13, 4363 (2023). 10.1038/s41598-023-31367-4 36928068 PMC10020546

[33] Pascal J. M. The comings and goings of PARP-1 in response to DNA damage. DNA Repair (Amst) 71, 177–182 (2018). 10.1016/j.dnarep.2018.08.022 30177435 PMC6637744

[34] Langelier M. F. PARP enzyme de novo synthesis of protein-free poly(ADP-ribose). Mol Cell (2024). 10.1016/j.molcel.2024.10.024 PMC11664634 39536748

[35] Okano S., Lan L., Caldecott K. W., Mori T. & Yasui A. Spatial and temporal cellular responses to single-strand breaks in human cells. Mol Cell Biol 23, 3974–3981 (2003). 10.1128/mcb.23.11.3974-3981.2003 12748298 PMC155230

[36] Okano S., Lan L., Tomkinson A. E. & Yasui A. Translocation of XRCC1 and DNA ligase IIIalpha from centrosomes to chromosomes in response to DNA damage in mitotic human cells. Nucleic Acids Res 33, 422–429 (2005). 15653642 10.1093/nar/gki190 PMC546168

[37] Caldecott K. W. XRCC1 protein; Form and function. DNA Repair (Amst) 81, 102664 (2019). 10.1016/j.dnarep.2019.102664 31324530

[38] Polo L. M. Efficient Single-Strand Break Repair Requires Binding to Both Poly(ADP-Ribose) and DNA by the Central BRCT Domain of XRCC1. Cell Rep 26, 573–581.e575 (2019). 10.1016/j.celrep.2018.12.082 30650352 PMC6334254

[39] Wilson S. H. & Kunkel T. A. Passing the baton in base excision repair. Nat Struct Biol 7, 176–178 (2000). 10700268 10.1038/73260

[40] Sallmyr A., Bhandari S. K., Naila T. & Tomkinson A. E. Mammalian DNA ligases; roles in maintaining genome integrity. J Mol Biol 436, 168276 (2024). 10.1016/j.jmb.2023.168276 37714297 PMC10843057

[41] Mackey Z. B. DNA ligase III is recruited to DNA strand breaks by a zinc finger motif homologous to that of poly(ADP-ribose) polymerase. Identification of two functionally distinct DNA binding regions within DNA ligase III. J Biol Chem 274, 21679–21687 (1999). 10419478 10.1074/jbc.274.31.21679

[42] Leppard J. B., Dong Z., Mackey Z. B. & Tomkinson A. E. Physical and functional interaction between DNA ligase IIIalpha and poly(ADP-Ribose) polymerase 1 in DNA single-strand break repair. Mol Cell Biol 23, 5919–5927 (2003). 12897160 10.1128/MCB.23.16.5919-5927.2003 PMC166336

[43] Cotner-Gohara E., Kim I. K., Tomkinson A. E. & Ellenberger T. Two DNA-binding and nick recognition modules in human DNA ligase III. J Biol Chem 283, 10764–10772 (2008). 10.1074/jbc.M708175200 18238776 PMC2447648

[44] Cannan W. J., Rashid I., Tomkinson A. E., Wallace S. S. & Pederson D. S. The Human Ligase IIIα-XRCC1 Protein Complex Performs DNA Nick Repair after Transient Unwrapping of Nucleosomal DNA. J Biol Chem 292, 5227–5238 (2017). 10.1074/jbc.M116.736728 28184006 PMC5392670

[45] Odell I. D. Nucleosome Disruption by DNA Ligase III-XRCC1 Promotes Efficient Base Excision Repair. Molecular and Cellular Biology 31, 4623–4632 (2011). 10.1128/MCB.05715-11 21930793 PMC3209256

[46] McNally J. R. & O’Brien P. J. Kinetic analyses of single-stranded break repair by human DNA ligase III isoforms reveal biochemical differences from DNA ligase I. Journal of Biological Chemistry 292, 15870–15879 (2017). 10.1074/jbc.M117.804625 28751376 PMC5612117

[47] Mackey Z. B. An alternative splicing event which occurs in mouse pachytene spermatocytes generates a form of DNA ligase III with distinct biochemical properties that may function in meiotic recombination. Mol Cell Biol 17, 989–998 (1997). 9001252 10.1128/mcb.17.2.989 PMC231824

[48] Sutton T. B. Global screening of base excision repair in nucleosome core particles. DNA Repair (Amst) 144, 103777 (2024). 10.1016/j.dnarep.2024.103777 39476546 PMC11611610

[49] Schaich M. A. Single-molecule analysis of DNA-binding proteins from nuclear extracts (SMADNE). Nucleic Acids Res 51, e39 (2023). 10.1093/nar/gkad095 36861323 PMC10123111

[50] McNally J. R. & O’Brien P. J. Kinetic analyses of single-stranded break repair by human DNA ligase III isoforms reveal biochemical differences from DNA ligase I. J Biol Chem 292, 15870–15879 (2017). 10.1074/jbc.M117.804625 28751376 PMC5612117

[51] Crut A., Nair P. A., Koster D. A., Shuman S. & Dekker N. H. Dynamics of phosphodiester synthesis by DNA ligase. Proc Natl Acad Sci U S A 105, 6894–6899 (2008). 10.1073/pnas.0800113105 18458338 PMC2383972

[52] Abdou I., Poirier G. G., Hendzel M. J. & Weinfeld M. DNA ligase III acts as a DNA strand break sensor in the cellular orchestration of DNA strand break repair. Nucleic Acids Res 43, 875–892 (2015). 10.1093/nar/gku1307 25539916 PMC4333375

[53] Mackey Z. B. An Alternative Splicing Event Which Occurs in Mouse Pachytene Spermatocytes Generates a Form of DNA Ligase III with Distinct Biochemical Properties That May Function in Meiotic Recombination. Molecular and Cellular Biology 17, 989–998 (1997). 10.1128/MCB.17.2.989 9001252 PMC231824

[54] Taylor R. M., Whitehouse C. J. & Caldecott K. W. The DNA ligase III zinc finger stimulates binding to DNA secondary structure and promotes end joining. Nucleic Acids Res 28, 3558–3563 (2000). 10.1093/nar/28.18.3558 10982876 PMC110727

[55] Luger K., Mäder A. W., Richmond R. K., Sargent D. F. & Richmond T. J. Crystal structure of the nucleosome core particle at 2.8 Å resolution. Nature 389, 251–260 (1997). 9305837 10.1038/38444

[56] Cannan W. J., Rashid I., Tomkinson A. E., Wallace S. S. & Pederson D. S. The Human Ligase IIIalpha-XRCC1 Protein Complex Performs DNA Nick Repair after Transient Unwrapping of Nucleosomal DNA. J Biol Chem 292, 5227–5238 (2017). 10.1074/jbc.M116.736728 28184006 PMC5392670

[57] Odell I. D. Nucleosome disruption by DNA ligase III-XRCC1 promotes efficient base excision repair. Mol Cell Biol 31, 4623–4632 (2011). 10.1128/MCB.05715-11 21930793 PMC3209256

[58] David S. S., O’Shea V. L. & Kundu S. Base-excision repair of oxidative DNA damage. Nature 447, 941–950 (2007). 10.1038/nature05978 17581577 PMC2896554

[59] Lowary P. T. & Widom J. New DNA sequence rules for high affinity binding to histone octamer and sequence-directed nucleosome positioning. J Mol Biol 276, 19–42 (1998). 10.1006/jmbi.1997.1494 9514715

[60] Díaz-Celis C. Assignment of structural transitions during mechanical unwrapping of nucleosomes and their disassembly products. Proc Natl Acad Sci U S A 119, e2206513119 (2022). 10.1073/pnas.2206513119 35939666 PMC9388122

[61] McGinty R. K. & Tan S. Principles of nucleosome recognition by chromatin factors and enzymes. Curr Opin Struct Biol 71, 16–26 (2021). 10.1016/j.sbi.2021.05.006 34198054 PMC8648869

[62] Duclos S., Doublié S. & Wallace S. S. in The Cellular Response to the Genotoxic Insult Issues in Toxicology (eds Greim Helmut & Albertini Richard) 115–159 (The Royal Society of Chemistry, 2012).

[63] Sallmyr A., Rashid I., Bhandari S. K., Naila T. & Tomkinson A. E. Human DNA ligases in replication and repair. DNA Repair (Amst) 93, 102908 (2020). 10.1016/j.dnarep.2020.102908 33087274 PMC8727047

[64] Tomkinson A. E. & Sallmyr A. Structure and function of the DNA ligases encoded by the mammalian LIG3 gene. Gene 531, 150–157 (2013). 10.1016/j.gene.2013.08.061 24013086 PMC3881560

[65] Chatterjee S. Probing the mechanism of nick searching by LIG1 at the single-molecule level. Nucleic Acids Res (2024). 10.1093/nar/gkae865 PMC11551761 39404052

[66] Tang Q. & Caglayan M. The scaffold protein XRCC1 stabilizes the formation of polbeta/gap DNA and ligase IIIalpha/nick DNA complexes in base excision repair. J Biol Chem 297, 101025 (2021). 10.1016/j.jbc.2021.101025 34339737 PMC8405949

[67] Cotner-Gohara E., Kim I.-K., Tomkinson A. & Ellenberger T. Two DNA-binding and nick recognition modules in human DNA ligase III. The Journal of biological chemistry 283, 10764–10772 (2008). 10.1074/jbc.M708175200 18238776 PMC2447648

[68] McNally J. R. Kinetic Analysis of Human DNA Ligase III Doctor of Philosophy thesis, University of Michigan, (2019).

[69] Dong Z. & Tomkinson A. E. ATM mediates oxidative stress-induced dephosphorylation of DNA ligase IIIalpha. Nucleic Acids Res 34, 5721–5279 (2006). 10.1093/nar/gkl705 17040896 PMC1694025

[70] Nosella M. L. Poly(ADP-ribosyl)ation enhances nucleosome dynamics and organizes DNA damage repair components within biomolecular condensates. Mol Cell 84, 429–446 e417 (2024). 10.1016/j.molcel.2023.12.019 38215753

[71] Wang M. D., Yin H., Landick R., Gelles J. & Block S. M. Stretching DNA with optical tweezers. Biophys J 72, 1335–1346 (1997). 10.1016/s0006-3495(97)78780-0 9138579 PMC1184516

